# Multi-drug resistance and reduced susceptibility to ciprofloxacin among *Salmonella enterica* serovar Typhi isolates from the Middle East and Central Asia

**DOI:** 10.1002/nmi2.46

**Published:** 2014-06-27

**Authors:** B A Rahman, M O Wasfy, M A Maksoud, N Hanna, E Dueger, B House

**Affiliations:** 1 Global Disease Detection and Response Program, U.S. Naval Medical Research Unit No. 3PSC 452 Box 5000, Cairo, FPO AE 09835-9998, Egypt; 2 Central Public Health LaboratoriesCairo, Egypt; 3 Centers for Disease Control and PreventionAtlanta, GA, 30333, USA

**Keywords:** Decreased ciprofloxacin susceptibility, fluoroquinolone resistance, multidrug-resistant typhoid, nalidixic acid resistance, *Salmonella* Typhi

## Abstract

Typhoid fever is common in developing countries, with an estimated 120 million infections and 700 000 annual deaths, worldwide. Fluoroquinolones have been the treatment of choice for infection with multidrug-resistant (MDR) *Salmonella enterica* serovar Typhi (*S*. Typhi). However, alarming reports of fluoroquinolone-resistance and failure of typhoid fever treatment have recently been published. To determine the proportion of *S*. Typhi isolates with reduced susceptibility to ciprofloxacin (RSC) from six countries in the Middle East and Central Asia, 968 *S*. Typhi isolates collected between 2002 and 2007 from Egypt, Uzbekistan, Pakistan, Qatar, Jordan and Iraq were tested for antibiotic susceptibility to five antibiotics using the disc-diffusion method. MDR was defined as resistance to amicillin, chloramphenicol and trimethoprim-sulfamethoxazole. The E-test was employed to determine the MIC of ciprofloxacin only. Nalidixic acid resistance was evaluated as a marker for RSC. Interpretations were made according to CLSI guidelines. MDR strains were considerably more prevalent in Iraq (83%) and Pakistan (52%) compared with the other countries studied (13–52%). Nearly all isolates were susceptible (99.7%) to ceftriaxone. RSC was detected in a total of 218 isolates (22%), mostly from Iraq (54/59, 92%), Uzbekistan (98/123, 80%), Qatar (23/43, 54%) and Pakistan (31/65, 47%). Many of these (21%) were also MDR. Use of nalidixic acid resistance as an indicator for RSC was 99% sensitive and 98% specific. This study reinforces the need for routine antimicrobial susceptibility surveillance of enteric fever isolates and close review of current therapeutic policies in the region.

## Introduction

Typhoid fever presents a major public health problem where safe drinking water and sanitation are inadequate. Endemic regions include developing countries in south-central and South East Asia and many parts of Africa and Latin America 1. The disease is caused by *Salmonella enterica* serovar Typhi (*S. *Typhi). In Egypt, population-based studies have shown an annual incidence of 13/100 000 persons in Belbis District in the Nile Delta 2 and 61/100 000 persons in Fayoum Governorate in the south 3. Likewise, thousands of Iraqis are affected each year, with 10–20% mortality rates due to limited access to fresh water and dumping of sewage into the rivers 4. Infection is also common in Jordan, but data and epidemiological studies from the Ministry of Health are limited and incidence is not well defined because of the lack of efficient reporting systems 5. In Pakistan, the incidence of typhoid fever is comparatively high (451/100 000), supporting the previous findings of Kothari *et al*. 6 who had claimed higher typhoid fever burden in Asia than Africa. Meanwhile, increasing rates of typhoid fever have been detected in Uzbekistan due to the deterioration of water treatment and distribution systems in the republics of the former Soviet Union 7. In Qatar, most infections are imported from the endemic Indian sub-continent and the Far East through expatriate workers and visitors 1.

In the past, *S*. Typhi infections were routinely treated with chloramphenicol, ampicillin, or trimethoprim-sulfamethoxazole, but multidrug-resistance (MDR) to these antibiotics started to emerge in 1990 8. In response, physicians in endemic areas shifted to fluoroquinolones or third-generation cephalosporins to ensure better treatment outcomes 9,10. Although this has resulted in a gradual return of strain sensitivity to chloramphenicol 9,11,12, fluoroquinolones are often still preferentially used to achieve better recovery rates 13.

Despite the recently reported susceptibility of *S. *Typhi isolates to ciprofloxacin by the disc-diffusion method, patients in many endemic areas have begun to present with clinical treatment failures leading to serious consequences 14–16. This treatment failure was observed for the first time in India in 1991 and subsequently recognized in other nearby countries. However, the minimum inhibitory concentrations (MICs) of these isolates indicated reduced susceptibility to ciprofloxacin (RSC) 14,17. Since routine use of MIC methods is expensive, resistance to nalidixic acid, the predecessor of the quinolone family, has been used alternatively as an indirect evidence of fluoroquinolone resistance 14,16.

In this study, MDR resistance to ampicillin, chloramphenicol and trimethoprim-sulfamethoxazole was determined for 968 *S*. Typhi isolates that were collected from the Middle East and Central Asia between 2002 and 2007. Decreased susceptibility to fluoroquinolones was also evaluated for the first time in Egypt and neighbouring countries using standard ciprofloxacin and nalidixic acid disc-diffusion tests.

## Materials and Methods

*Salmonella* Typhi blood culture isolates collected during acute febrile illness surveillance in Egypt and Uzbekistan were archived at −70°C at the U.S. Naval Medical Research Unit No. 3 (NAMRU-3) and used for the purposes of this study. Isolates from other countries were recovered from stool or blood cultures of patients during sporadic acute febrile illness outbreaks in Iraq, Jordan, Pakistan and Qatar. Ethical approval for all sample collection was obtained from the institutional review boards of NAMRU-3 and the respective authorities in the collaborating countries. All study subjects provided written informed consent for participation.

Definitive identification was attained using the API 20E kit (bioMérieux, Marcy l'Etoile, France) and commercial antisera (BD Biosciences, Frankland Lakes, NJ, USA). Susceptibility to antibiotic discs containing ampicillin (10 mg), chloramphenicol (30 mg), trimethoprim-sulfamethoxazole (25 mg), ciprofloxacin (5 mg), ceftriaxone (30 mg) and nalidixic acid (30 mg) (Becton-Dickinson, Sparks, MD, USA) was evaluated using the disc-diffusion method. The E-test (bioMérieux) was employed to determine the MICs of ciprofloxacin only. All interpretations were made according to CLSI, 2012. MDR was defined as the simultaneous resistance of bacteria to chloramphenicol, ampicillin and trimethoprim-sulfamethoxazole. The sensitivity and specificity of the nalidixic acid assay as a surrogate of the ciprofloxacin E-test were calculated. Proportions were compared using the chi-squared test.

## Results

A total of 968 *S*. Typhi isolates from Egypt (*n* = 654), Uzbekistan (*n* = 123), Pakistan (*n* = 65), Iraq (*n* = 59), Qatar (*n* = 43) and Jordan (*n* = 24) were used in this study. The prevalence of MDR *S*. Typhi isolates was significantly higher in Iraq (49/59, 83%) and Pakistan (34/65, 52%) than in other countries included in this study (13–17%, p <0.01; Table1). Within Egypt, the majority of isolates were from four different governorates: Fayoum (41%), Cairo (25%), Aswan (10%) and Alexandria (7%). Fayoum isolates showed a significantly higher MDR prevalence (29%; p <0.05) when compared with the other governorates (0–7%; p <0.05; Table2).

**Table 1:** Antimicrobial disc-diffusion resistance profiles of 968 *Salmonella enterica* serotype Typhi isolates collected from six countries between 2002 and 2007 (approximate percentages per country are given in parentheses)

Country	No. of isolates	C (%)			AM (%)			CRO (%)			CIP (%)			SXT (%)			NA (%)			MDR (%)
		S	I	R	S	I	R	S	I	R	S	I	R	S	I	R	S	I	R	
Egypt	654	554 (85)	0	100 (15)	555 (85)	2 (<1)	97 (15)	652 (100)	2 (<1)	0	421 (64)	229 (35)	4 (<1)	552 (85)	3 (<1)	99 (15)	618 (94)	26 (4)	10 (2)	94 (14)
Iraq	59	8 (14)	0	51 (86)	7 (12)	0	52 (88)	59 (100)	0	0	11 (19)	38 (64)	10 (17)	9 (15)	0	50 (85)	5 (8)	0	54 (92)	49 (83)
Jordan	24	18 (75)	0	6 (25)	20 (83)	0	4 (17)	24 (100)	0	0	9 (37)	15 (63)	0	20 (83)	0	4 (17)	18 (75)	4 (17)	2 (8)	4 (17)
Pakistan	65	30 (46)	0	35 (54)	31 (48)	0	34 (52)	65 (100)	0	0	10 (15)	39 (60)	16 (25)	29 (45)	0	36 (55)	29 (45)	5 (8)	31 (47)	34 (52)
Qatar	43	37 (86)	0	6 (14)	37 (86)	0	6 (14)	43 (100)	0	0	15 (35)	21 (49)	7 (16)	37 (86)	0	6 (14)	20 (46)	0	23 (54)	6 (14)
Uzbekistan	123	106 (86)	0	17 (14)	100 (81)	0	23 (19)	123 (100)	0	0	11 (9)	104 (84)	8 (7)	106 (86)	0	17 (14)	24 (19)	1 (1)	98 (80)	16 (13)
Total	968	753 (78)	0	215 (22)	750 (77)	2 (<1)	216 (23)	966 (100)	2 (<1)	0	468 (48)	455 (47)	45 (5)	753 (78)	3 (<1)	212 (22)	714 (74)	36 (4)	218 (22)	203 (21)

C, chloramphenicol; AM, ampicillin; CRO, ceftriaxone; CIP, ciprofloxacin; SXT, trimethoprim-sulfamethoxazole; NA, nalidixic acid; MDR, multidrug resistance for C, AM and SXT.

**Table 2:** Antibiotic disc-diffusion profiles of 654 *Salmonella enterica* serotype Typhi isolates collected between 2002 and 2007 from major governorate infectious disease ‘Fever’ hospitals in Egypt (approximate percentages per governorate are given in parentheses)

Governorate	No. of isolates	C (%)			AM (%)			CRO (%)			CIP (%)			SXT (%)			NA (%)			MDR (%)
		S	I	R	S	I	R	S	I	R	S	I	R	S	I	R	S	I	R	
Fayoum	270	188 (70)	0	82 (30)	191 (71)	0	79 (29)	270 (100)	0	0	152 (56)	118 (44)	0	189 (70)	0	81 (30)	254 (94)	13 (5)	3 (1)	77 (29)
Cairo	166	154 (93)	0	12 (7)	154 (93)	0	12 (7)	166 (100)	0	0	117 (70)	49 (30)	0	154 (93)	0	12 (7)	158 (95)	6 (4)	2 (1)	12 (7)
Aswan	66	65 (98)	0	1 (2)	65 (98)	0	1 (2)	66 (100)	0	0	47 (71)	19 (29)	0	64 (97)	0	2 (3)	64 (97)	1 (1)	1 (1)	1 (2)
Alexandria	46	45 (98)	0	1 (2)	43 (94)	2 (4)	1 (2)	44 (96)	2 (4)	0	32 (70)	13 (28)	1 (2)	44 (96)	1 (2)	1 (2)	43 (92)	2 (4)	2 (4)	1 (2)
Gharbiya	40	36 (90)	0	4 (10)	36 (90)	0	4 (10)	40 (100)	0	0	21 (52)	18 (45)	1 (3)	37 (92)	0	3 (8)	37 (92)	2 (5)	1 (3)	3 (8)
Menofiya	12	12 (100)	0	0	12 (100)	0	0	12 (100)	0	0	10 (83)	2 (17)	0	12 (100)	0	0	10 (83)	2 (17)	0	0
Asiut	26	26 (100)	0	0	26 (100)	0	0	26 (100)	0	0	23 (88)	1 (4)	2 (8)	24 (92)	2 (8)	0	26 (100)	0	0	0
Others^a^	27	27 (100)	0	0	27 (100)	0	0	27 (100)	0	0	19 (70)	8 (30)	0	27 (100)	0	0	26 (96)	0	1 (4)	0

C, chloramphenicol; AM, ampicillin; CRO, ceftriaxone; CIP, ciprofloxacin; SXT, trimethoprim-sulfamethoxazole; NA, nalidixic acid; MDR, multidrug resistance for C, AM and SXT.

a Includes ‘Fever’ hospitals in Sharquia, Port Said, Quena and Sohag governorates.

Nearly all isolates were susceptible to ceftriaxone, except for two from Alexandria, Egypt, which showed intermediate resistance (total susceptibility 99.7%) and were negative for extended spectrum β-lactamase production. For ciprofloxacin, only 48% were susceptible by the disc-diffusion method, with zone diameters ≥31 mm (Table1). The remaining isolates were either intermediate (47%, 21–30 mm) or fully resistant (5%, ≤20 mm), with relatively elevated MICs ranging from 0.125 to 0.75 μg/mL (mean 0.33 ± 0.12 μg/mL). Meanwhile, susceptibility to nalidixic acid by the disc-diffusion method was 74%, 4% were intermediate and 22% were resistant. Isolates that tested nalidixic acid resistant (*n* = 218, 22%) correspondingly showed elevated MICs for ciprofloxacin in 216 isolates, ranging from 0.125 to 0.94 μg/mL (average 0.29 ± 0.11 μg/mL) RSC. The remaining two isolates were susceptible to ciprofloxacin (<0.064 μg/mL). The distribution of RSC reflected a broad geographic variability: Iraq (92%); Uzbekistan (80%); Qatar (54%); Pakistan (47%); Jordan (8%) and; Egypt (2%) (Table1). Use of nalidixic acid resistance as an indicator of reduced ciprofloxacin susceptibility was 99% sensitive and 99.7% specific.

## Discussion

The high proportions of *S. *Typhi strains demonstrating MDR from Iraq (83%) and Pakistan (52%) relative to those from other countries in this study (≤17%) are consistent with a recent report indicating that 80% of patients infected with MDR *S*. Typhi originate from the Asian continent and the remainder occur mostly in Africa and Latin America 18. Self-medication and unguided antibiotic treatment are two main reasons for MDR development. Since the institution of ciprofloxacin as a preferred treatment choice for MDR *S. *Typhi cases, RSC has emerged, first in India (69%) in 1996, Vietnam (76%) in 1997, Tajikistan (the percentage is not clear) in 1998 and subsequently in other parts of the world, including the UK, Mexico, Thailand, Korea and Peru 19,20. In our study the prevalence of RSC was predictable in Pakistan (47%), but the particularly high rate (92%) seen in Iraq may indicate a widespread and/or inappropriate use of fluoroquinolones in this country. This may concur with the observation that many isolates tested from this region were obtained during acute febrile illness outbreaks, which is probably a limitation that reflects the selection of more serious or more resistant epidemic strains.

Although only 13% of Uzbekistan isolates from this study were MDR, 80% showed RSC, in agreement with previous findings from Samarkand, the second largest city in Uzbekistan 7. This may indicate that fluoroquinolones have been used more commonly than other conventional antibiotics for the treatment of typhoid fever. In 2003, the government of Uzbekistan implemented routine immunizations in specific areas of the country to reduce typhoid fever incidence 21.

In Qatar, over one half of the isolates demonstrated RSC (54%), while only 14% were MDR. This may be attributed to the importation of *S*. Typhi strains from the Indian sub-continent, where it is endemic, and the Far East; it has been estimated that expatriate workers constitute 30–40% of the population in the Gulf States 1. A recent study 22 detected RSC in 44% of *S*. Typhi isolates from the Gulf region and warned against compromising treatments.

In Amman, Jordan, 8% of isolates demonstrated RSC, a relatively small percentage that is contrary to a recent local report claiming virtually no resistance to this drug among *S*. Typhi isolates between 2004 and 2006 5. The discrepancy in Jordan's findings may be attributed to the involvement of sporadic cases living in the Jordan Valley, about 50 km west of Amman, disconnecting this area from incoming strains to the country's capital through foreign labor.

In Egypt, the rate of RSC among *S*. Typhi isolates was low (2%), ranging from 0% in Asiut Governorate (in southern Egypt), Menofyia (Nile Delta region), Gharbia (3%, Delta) to 4% in Alexandria (on the Mediterranean coast) (Table2). Similarly, MDR rates were low except in the Fayoum Governorate (29%; p <0.05), possibly as the result of poor sanitation, limited urbanization and unguided use of antibiotics in this governorate.

Our findings showed that almost all isolates were susceptible (99.7%) to ceftriaxone (a third-generation cephalosporin), which remains the drug of choice for the treatment of typhoid fever 9,10. However, the unprecedented detection of two isolates with intermediate resistance to ceftriaxone from Alexandria, Egypt is an early warning sign indicating the need for more controlled use of this drug in the country and in the region. Previously, all *S*. Typhi isolates from Egypt were claimed to be susceptible 10,23.

The continued spread of MDR strains and reduced effectiveness of standard drug regimens are serious threats to the treatment of typhoid fever in endemic countries. Although ceftriaxone was the most reliable choice for MDR and nalidixic acid-resistant *S*. Typhi isolates, a high level resistance to ceftriaxone (MIC 64 mg/L) has been reported from Bangladesh in 1999 24,25. The two isolates showing intermediate resistance to ceftriaxone in this study were negative for extended spectrum β-lactamase production, though reported in a strain from a 54 year old Dutch man returning from the Philippines 26.

The observation that nalidixic acid resistance can predict RSC in antimicrobial disc-diffusion susceptibility testing has been documented before 14,16. In this study, the sensitivity and specificity of the assay were 99% and 99.7%, respectively. It relies on a mutation in the *gyrA* gene and occasionally the *parC* gene is included, leading to higher level of resistance (MICs from 8 to >32 μg/mL) 27,28.

This study used 968 S. Typhi isolates collected from six countries over 6 years. The obtained data must be interpreted with care, as isolates may not be necessarily representative of either geographical coverage or time-frame. However, the findings alert the scientific and medical communities in the Middle East and Central Asia regions to the emergence of reduced fluoroquinolone susceptibility. The positive and negative predictive values of nalidixic acid susceptibility testing are high (99% and 99.7%, respectively) and present a cheaper and more practical tool to predict RSC. Consideration should be given to the use of more conventional drugs (chloramphenicol, ampicillin or trimethoprim-sulfamethoxazole) for the treatment of typhoid fever in countries with low MDR prevalence. Also, routine antibiotic susceptibility testing of isolates should be consistently performed (e.g. nalidixic acid testing to predict RSC) before prescribing quinolones or cephalosporins to avoid compromising treatment options.

## Copyright Assignment Statement

All authors except Dr Nagwa Hanaa are employees of the U.S. Government. This work was prepared as part of our official duties. Title 17 U.S.C. §105 provides that ‘Copyright protection under this title is not available for any work of the United States Government.’ Title 17 U.S.C. §101 defines a U.S. Government work as a work prepared by a military service member or employee of the U.S. Government as part of that person's official duties.
